# Unveiling transient protein-protein interactions that modulate inhibition of alpha-synuclein aggregation by beta-synuclein, a pre-synaptic protein that co-localizes with alpha-synuclein

**DOI:** 10.1038/srep15164

**Published:** 2015-10-19

**Authors:** Maria K. Janowska, Kuen-Phon Wu, Jean Baum

**Affiliations:** 1 Department of Chemistry and Chemical Biology, Rutgers University, Piscataway, New Jersey 08854

## Abstract

Pathology in Parkinson’s disease is linked to self-association of α-Synuclein (αS) into pathogenic oligomeric species and highly ordered amyloid fibrils. Developing effective therapeutic strategies against this debilitating disease is critical and βS, a pre-synaptic protein that co-localizes with αS, can act as an inhibitor of αS assembly. Despite the potential importance of βS as an inhibitor of αS, the nature, location and specificity of the molecular interactions between these two proteins is unknown. Here we use NMR paramagnetic relaxation enhancement experiments, to demonstrate that βS interacts directly with αS in a transient dimer complex with high specificity and weak affinity. Inhibition of αS by βS arises from transient αS/βS heterodimer species that exist primarily in head- to- tail configurations while αS aggregation arises from a more heterogeneous and weaker range of transient interactions that include both head-to-head and head-to-tail configurations. Our results highlight that intrinsically disordered proteins can interact directly with one another at low affinity and that the transient interactions that drive inhibition versus aggregation are distinct by virtue of their plasticity and specificity.

Protein aggregation is the origin of a wide variety of human neurodegenerative diseases including Parkinson’s, Alzheimer’s, Huntington’s and Creutzfeldt-Jakob disease^1^,^2^. Parkinson’s disease (PD), a highly debilitating illness, is the second most prevalent of the late onset neurodegenerative diseases and affects as many as 6 million people worldwide^3^. Αs life expectancy continues to increase, neurodegenerative diseases like Parkinson’s are becoming increasingly common and a threat to global public health. The diagnostic hallmark of PD is a deposit called a Lewy body that is primarily composed of the pre-synaptic intrinsically disordered protein α-synuclein (αS). Fibril formation of αS is also implicated in other neurodegenerative diseases, including multiple system atrophy and dementia with Lewy bodies, referred to as synucleinopathies. Although the function of αS is not clearly defined it is thought to be involved in promoting SNARE complex assembly^4^,^5^, in regulation of the synaptic vesicle pool^6^,^7^, and in remodeling membranes^8^. The origin of αS pathology in neurodegenerative disease is clearly linked to the self-association of the intrinsically disordered αS monomers into pathogenic oligomeric species and highly ordered amyloid fibrils.

One approach to developing effective therapeutic strategies against this debilitating disease is to identify inhibitors of αS aggregation. Small molecule inhibitors of αS have been proposed^9^,^10^,^11^,^12^,^13^. In addition, proteins such as heat shock proteins (Hsp40, Hsp70, Hsp90, αB-crystallin)^14^,^15^, and the intrinsically disordered β-synuclein (βS), a homologue of αS with which it co-localizes have been shown to interfere with αS assembly. A number of studies have established a neuroprotective role for βS^16^,^17^,^18^,^19^,^20^,^21^,^22^,^23^,^24^. Masliah and co-workers have shown that βS is expressed at similar levels as to αS in the central nervous system. However the ratio of βS to αS at the mRNA level is significantly decreased in diseased brains, suggesting a regulatory role within the synuclein family^24^. *In vivo* it has been shown that over-expression of αS with βS in mouse models significantly decreases the number of plaques formed^25^ and that intracerebral injection of the lenti-βS virus reduces the formation of αS inclusions in transgenic mice^26^. *In vitro*, it has been shown that the presence of βS with αS slows its aggregation^16^,^17^,^18^,^25^,^26^,^27^. Despite the fact that βS has a very similar sequence to αS, it does not form fibrils on its own^16^,^28^,^29^,^30^, but may form aggregates whose toxicity is debated^31^,^32^. The *in vivo* data clearly suggest that βS plays an important regulatory role in inhibition of αS pathology but at this stage there is no molecular information about the nature, location and specificity of the protein-protein interactions that initiate the inhibition of αS by βS.

To understand the mechanism by which the intrinsically disordered protein βS interacts with αS, we use NMR to map the monomer-monomer interactions that lead to inhibition or promotion of aggregation. Despite the importance of these interactions, the molecular details are extremely difficult to obtain due to their transient nature and low population. Paramagnetic relaxation enhancement experiments (PRE) offer an excellent tool for characterization of weak and transient interactions because they are able to probe states that exist at low populations (even 0.5–5%) and exhibit short life times (250–500 μs)^33^,^34^. Here we use inter-chain NMR PRE experiments to identify and characterize weak transient complexes of αS and βS^35^. We show that αS homo-dimers sample a heterogeneous range of population distributions, including head- to- head and head- to- tail configurations, while αS/βS hetero-dimers exist primarily in head- to- tail configurations.

NMR inter-chain PRE titration experiments previously used on folded proteins^36^ are applied here to intrinsically disordered proteins. These experiments allow us to obtain residue specific dissociation constants to inform us about the specificity and affinity of dimer interactions in the different regions of the transient disordered complexes. Our results show that the hetero-dimer transient head- to- tail interactions between αS and βS are approximately 5 times stronger than the interactions observed in the homo-dimer αS species suggesting that these αS/βS interactions create a kinetic trap which delays or inhibits the formation of αS fibrils. The novel insight presented in this paper not only defines residue specific contacts between two intrinsically disordered proteins but also links the homo and hetero-complexes with distinct pathways that lead to aggregation versus inhibition.

## Results

### βS inhibits αS fibril formation in a dose dependent manner

**Figure 1:**
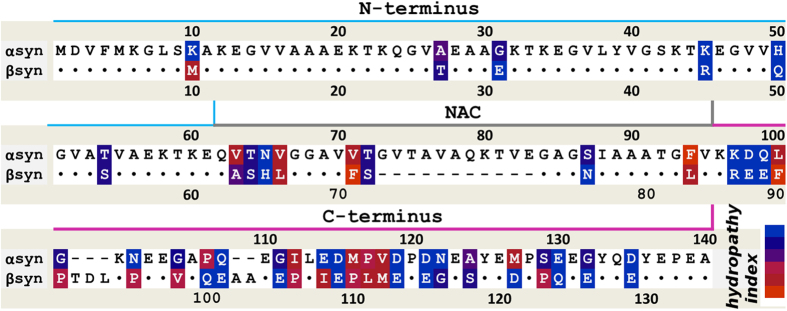
Sequence alignment of αS and βS shows high similarity between proteins. Sequence differences are color-coded according to the hydrophobicity index shown in the bottom right (red-hydrophobic, blue-hydrophilic). Identical residues are shown by dots and deletions are shown by dashes. The line above the sequence shows the N-(blue), NAC (grey) and C-terminal regions (pink).

αS and βS are part of the synuclein family. These can be described as: the N-terminus that contains KTKXGV repeats and forms helices at membranes^37^, the non-amyloid-βS component (NAC) region, and the highly acidic and solubilizing C-terminus (Fig. 1). αS and βS have similar sequences, particularly at the N-terminus, but very different fibrillation and oligomerization properties (Fig. 1). The N-terminus for all synucleins is highly conserved, with only 6 substitutions between αS and βS sequences. In contrast, the C-terminus is the least conserved region with more prolines and more negatively charged residues with a net charge of -12 in αS and -15 in βS. βS has an 11 residue deletion in the NAC region that was thought to be important in preventing fibril formation but substitution of this region into βS does not recover full fibrillation potential of αS^28^,^29^.

Similarly to αS it has been established that the physiological form of βS and its pathological mutants *in vivo* are N-terminally acetylated^38^. All experiments in this study are performed on the acetylated forms of the protein that we will refer to as αS and βS. All previous characterization of βS was performed on non-acetylated protein^16^,^17^,^28^,^39^, therefore we use NMR and other biophysical approaches here to determine whether N-terminal acetylation affects the conformation or oligomerization state of the protein. NMR and other biophysical techniques including dynamic light scattering (DLS) and circular dichroism (CD) show that acetylated βS, similarly to acetylated αS, is primarily monomeric and unfolded (Supplementary Fig. 1 & 2). Secondary structure propensities indicate the formation of a transient N-terminal helix relative to the non-acetylated form of βS similarly to αS (Supplementary Fig. 1 & 2). In addition, the C-terminus of acetylated βS is more extended that the C-terminus of acetylated αS consistent with previous results on non-acetylated protein^40^. Electrospray ionization mass spectroscopy (ESI-MS) experiments (Supplementary Fig. 1) show that acetylated βS can populate an extended and a compact form with a higher population of extended conformation relative to acetylated αS.

**Figure 2:**
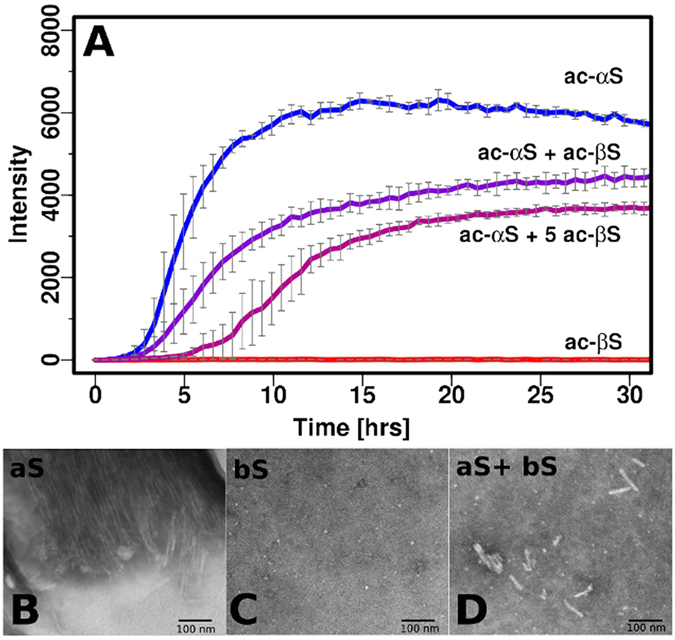
Aggregation inhibiton of αS by βS as monitored by ThT and TEM. (**A**) ThT fibrillation assay curves of: αS alone (blue), βS alone (blue), αS+βS 1:1 mixture (violet), αS+βS 1:5 mixture (magenta) Experimenal conditions: 10 mM MES, pH 6, 70 μM protein. The experiment was performed in triplicate at 37 °C with shaking with teflon beads and average values were plotted with associated error bars. The buffer conditions matched the conditions of NMR PRE expreriments. Negatively stained electron micrographs (scale 100 nm) of end products of the ThT fibrillation assay of: (**B**) αS (**C**) βS (**D**) αS co-incubated with βS, ratio 1:1.

Addition of acetylated βS to acetylated αS inhibits fibril formation in a dose dependent manner, consistent with previous findings for the non-acetylated forms of the proteins (Fig. 2A)^16^. As the concentration of βS is increased we observe a significant change in the rate of the elongation phase and in the total ThT intensity as well as a small change in the lag phase. Previous work has shown that ThT can be used as a semiquantitative method to estimate relative amounts of fibril formed, although caution should be applied in interpreting ThT intensities^41^,^42^. In this case, the inhibition of fibril formation as shown by the ThT experiments is supported by Transmission Electron Microscopy (TEM) data which shows significant changes in fibril morphology of αS/βS relative to fibrils of αS alone (Fig. 2B–D). βS does not form fibrils and the αS/βS mixture forms significantly fewer and shorter fibrils than αS alone as seen in TEM (Fig. 2C,D).

### Mapping of residue specific transient interactions in αS/αS homo- and αS/βS hetero-dimer complexes using NMR inter-chain PRE experiments

ThT fluorescence experiments and TEM data (Fig. 2) have established that βS alters the aggregation kinetics of αS, however there has been no evidence to date of a direct interaction between these proteins. To determine the existence of, and to characterize transient inter-chain interactions between homo-dimers (αS/αS, βS/βS) and hetero-dimers (αS/βS, βS/αS), inter-chain NMR paramagnetic relaxation enhancement experiments were performed. In this experiment, NMR blind ^14^N-MTSL labeled protein is mixed with NMR visible (^15^N) unmodified protein and broadening of signal is limited to residues on the NMR visible chain that interact with the MTSL labels on the NMR blind chain (Supplementary Fig. 3). Signal broadening therefore reflects interactions between the NMR blind and NMR visible protein^33^,^34^,^35^,^43^.

**Figure 3:**
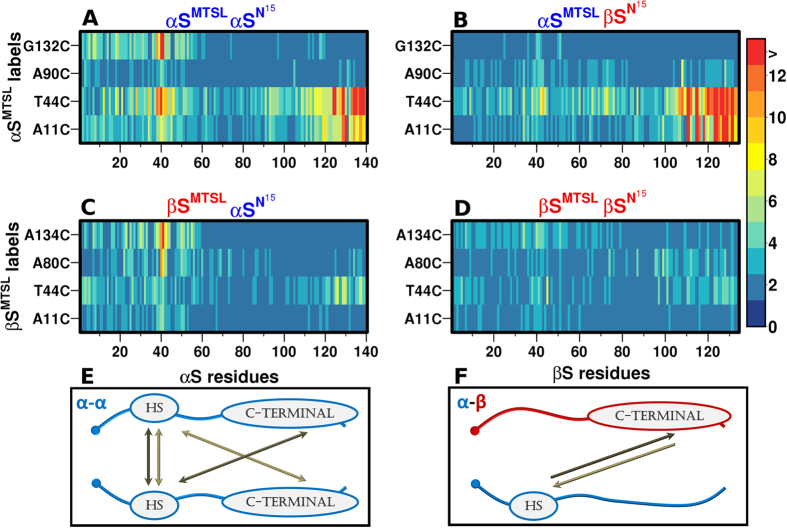
Contact maps of αS/αS homo-dimers and αS/βS hetero-dimers show distinctive interaction profiles. Contact maps of transient dimers, shown as heat maps where each strip represents the color coded value of the residue-specific inter-chain paramagnetic relaxation enhancement rate (PRE rate-H_N_Γ_2_) induced by the proximity of the MTSL label to the residues in the indicated protein. Contact maps (**A**–**D**): figures A-D show four heat maps for all possible permutations of the spin label and NMR detectable chains of αS and βS: (**A**) ^14^N-αS-MTSL/^15^N-αS, (**B**) ^14^N-αS-MTSL/^15^N-βS, (**C**) ^14^N-βS-MTSL/^15^N-αS, (**D**) ^14^N-βS-MTSL/^15^N-βS. Contact maps show the PRE values colored in accordance with the legend; residues that do not exhibit interactions are colored blue and interactions higher than 12 Hz are colored red. Each strip on the contact maps corresponds to the spin label (y-axis). In one strip there are bins which correspond to the residue number on the NMR visible chain (x-axis). (**E**,**F**) Schematic representations of possible interactions of homo αS/ αS and hetero- αS/βS synuclein dimers. (**E**) αS/αS corresponds to interactions from the contact map (**A**,**F**) αS/βS corresponds to contact map (**B**,**C**); no homo-dimer for βS is shown as we observe no inter-chain interactions between βS chains. HS in the schematic stands for interactive “Hot Spot”. The hot spot size is defined based on the interactivity of the regions as shown in Supplementary Fig. 4A. Hot spot encompasses residues 38–45.

NMR PRE experiments were performed on all four combinations of possible homo- and hetero-dimers (αS^15N^αS^MTSL^, αS^15N^βS^MTSL^, βS^15N^αS ^MTSL^, βS^15N^βS^MTSL^) to determine whether there is evidence for direct residue specific transient interactions and to establish the location of the interactions. The results of Fig. 3 are striking as they show immediately that the four combinations of possible hetero and homo-dimers have different transient interaction profiles (Fig. 3 and Supplementary Fig. 4). There is evidence for interactions between the homo-dimer complexes of αS, and the hetero-dimer complexes of αS and βS; interactions between βS are essentially non-existent. We have introduced four spin labels along the sequence at positions 11, 44, 90 and 132 for αS and 11, 44, 80, 134 for βS to probe interactions in the N, NAC and C-terminal regions. Experimental results are presented as heat maps (Fig. 3A–D), where each strip shows color-coded values of residue-specific inter-chain paramagnetic relaxation enhancement rates (PRE rate-Γ) induced by the proximity of the MTSL label to another chain. Strong interactions (>12 Hz) are defined relative to the lack of interactions otherwise observed in the βS homo-dimer contact maps. Under the conditions of the experiment, αS and βS do not form fibrils or oligomers; therefore dimer detection arises as a result of low populations of dimers existing in equilibrium with the monomer precursor (for αS lack of higher order species in the sample has been confirmed by ESI-IMS-MS experiments^44^ and for hetero-species this has been confirmed with ESI-MS.

### αS populates a heterogeneous range of transient complexes while αS/βS hetero-complexes sample primarily head-to–tail non-propagating interactions

The heat map for the αS/αS homo-dimer shows strong interactions between the N-terminal αS-MTSL labeled positions A11 and T44 with the N-terminal region 36 to 44, and the C-terminal region 124 to 140 (Fig. 3A). Earlier studies by our group^35^ on αS showed transient inter-chain interactions between the N- and C- termini (N-C), but by increasing the number and positions of the MTSL spin labels we now observe new inter-chain interactions between the N-termini showing that αS can populate multiple dimer configurations. In noticeable contrast, the αS/βS hetero-dimers show strong interactions between the N-terminal αS-MTSL labeled positions A11 and T44 and the C-terminus from residues 105 to 134 and extremely weak N-N terminal interactions between 37 and 41 (Fig. 3B). According to the heat map, the hetero-dimer interactions between the N-terminus of αS and the C-terminus of βS appear to be more extensive and stronger (residues 105-134 compared to residues 124-140 in αS/αS homo-dimers) (Fig. 3A–C) than those in αS. βS shows extremely minimal interactions with itself supporting the view that βS does not form fibrils (Fig. 3D).

A schematic representation of the dominant homo and hetero-dimer interactions shows that both N-N and N-C configurations are sampled by the αS homo-dimers while only N-C terminal interactions are sampled in the αS/βS hetero-dimers (Fig. 3E,F). The favorable interactions of the N-terminal hydrophobic region of αS with itself (N-N), and with the C-terminus of αS and βS suggest that they act as an aggregation initiation region. The highly interactive N-terminal region encompassing residues 38- 45 is referred to as ‘hot spot’ region (Suppl. 4A). In the αS homo-complexes, the interactions detected by N- and C-terminal probes show symmetry, implying that the interactions we observe are not experimental artifacts. The NMR PRE data show that αS homo-dimers can sample a heterogeneous range of populations, including head- to- head and head- to- tail configurations while αS/βS hetero-dimers sample only head- to- tail dimers. This suggests that the hetero-dimers have a more limited range of conformational preferences for the dimer species.

### Aggregation versus inhibition of αS by βS is due to a balance between specificity and affinity of transient interactions

**Figure 4:**
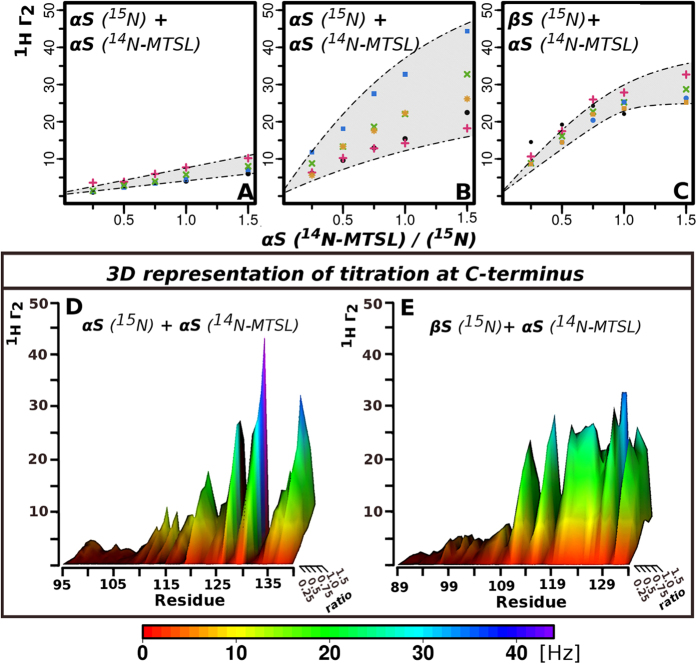
Residue specific binding affinities for transient αS/βS hetero-complexes show higher specificity and affinity than αS/αS homo-complexes. (**A**–**C**) Representative examples of titration curves arising from PRE titration profiles from αS-44-MTSL to ^15^N-αS and ^15^N-βS. Illustration of how the K_D_ is obtained from fitting the PRE titration data as a function of the molar ratio of ^14^N-αS-T44C-MTSL labeled to ^15^N-αS (**A**,**B**) and to ^15^N-βS (**C**). (**A**) αS residues 38–41 show a linear dependence between PRE values and concentration indicating non-specific interactions at these sites, (Residues colors: 38-black, 39- blue, 40-pink, 41-green), (**B**) αS residues 125–140 exhibit non-linear titration curves indicating specific interactions. (Residues colors: 125-orange, 130-blue, 135-pink, 137-green, 139-black), (**C**) βS residues 109–134 interact strongly and more uniformly with αS-44-MTSL (Residues colors: 115-green, 120-orange, 121-blue, 129-pink, 131-black). Shaded areas indicate the range of possible K_D_ values. Titration profiles for αS/βS show a higher degree of saturation consistent with more specificity and higher affinity than those for αS/ αS (**D**,**E**) 3D representation of titration curves in the C-terminus (**D**) ^15^N-αS C-terminus titrated with T44C-MTSL labeled ^14^N-αS (**E**) ^15^N-βS C-terminus titrated with T44C-MTSL labeled ^14^N-αS titration. The x-axis shows the C-terminal residues of both αS and βS after sequence alignment, the z- axis depicts the PRE values, the y- axis the ratio of T44C-MTSL labeled ^14^N-αS to ^15^N sample concentration. The surface is colored using a rainbow palette, where low H_N_Γ_2_ values are red and the highest values are purple (according to legend below the plot).

The inter-chain NMR PRE experiments described above are powerful as they provide us with direct evidence for the existence of transient interactions and allow us to pinpoint the specific residues involved in these encounter complexes, however the specificity and affinity of the interactions remains unknown. We extend an earlier approach^36^,^45^ designed for folded proteins to transient encounter complexes of IDPs. We obtain residue-specific equilibrium dissociation constants (*K*_*D*_) by performing titrations of ^15^N labeled αS with MTSL-labeled ^14^N-αS. K_*D*_s are obtained by fitting the intermolecular transverse ^1^H relaxation rates to a titration curve (see methods) (Fig. 4).

NMR PRE titration experiments and data analysis is complex as K_*D*_s are anticipated to be weak due to the disordered nature of the monomers. The titration curves are grouped according to their profiles and three different types of patterns emerge (Fig. 4A–C). Representative examples of titration curves arising from interactions between αS-44-MTSL and residues 38–41 of the N-terminus (Fig. 4A), and residues 125–140 of the C-terminus (Fig. 4B), show that they are distinct from one another. The titration curves for the N-N interactions show a linear dependence between PRE values and concentration indicating non-specific interactions at this site, while interactions with the C-terminal 125–140 exhibit non-linear titration curves suggesting specific interactions. The range of K_D_ values in this group is between K_D_ ~ 500 μM (range 90–1200 μM) using the data analysis described in methods. While we observe both non-specific (N-N) (Fig. 4A) and specific interactions (N-C) (Fig. 4B) in homo-αS complexes, the hetero-complexes αS/βS (Fig. 4C) have only specific N-C interactions. The titration profiles of αS-44-MTSL with βS in the region 115–134 have a narrower range of K_D_ values (K_D_ ~ 100 μM, range 40–350 μM) than those associated with the K_D_ values of αS-44-MTSL αS with its own C-terminus (K_D_ ~ 500 μM, range 90–1200 μM) suggesting more uniform behavior across this region and higher specificity and affinity by approximately 5 fold (Supplementary Tables 1&2). The differences between the strengths of the interactions and the range of residues over which the interactions are occurring is seen clearly in the 3D plots where the interaction regions in αS are more rugged while the interactions between αS and βS are smoother, more uniform and extend over a wider range of residues (Fig. 4D,E).

## Discussion

**Figure 5:**
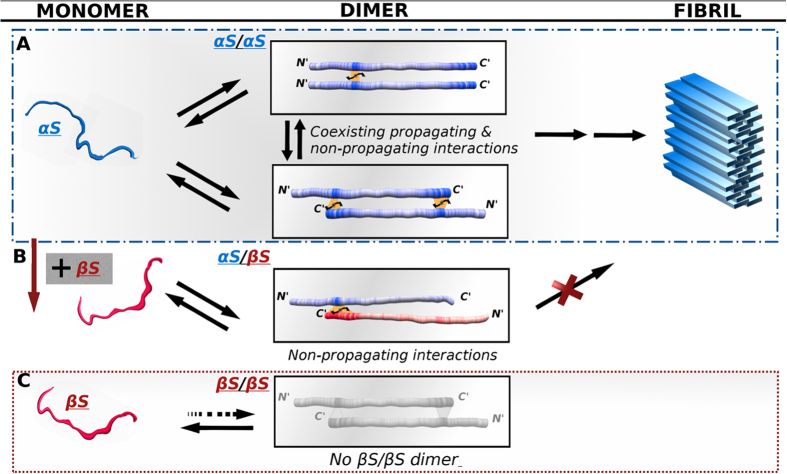
Schematic model of αS/αS and αS/βS transient interactions suggest a new molecular view of inhibition routes to αS aggregation. (**A**) αS/αS homo-dimer interactions: head-to-head aggregation prone interactions at residues 36–44 co-exist with head-to-tail inhibitory interactions at residues 125–140. (**B**) αS/βS hetero-dimer interactions: head- to- tail inhibitory interactions span a broader range of C-terminal residues 115–134 with higher affinity. (**C**) No interactions are observed between monomer chains of βS. Protein chains are color-coded according to the interactivity of the regions as shown in Supplementary Fig. 4. The NMR data show that αS homo-dimers can sample a heterogeneous range of poulations including N-to-N and N- to C-terminal configurations while αS/βS hetero-dimers sample only N- to C- terminal configurations with higher affinity and specificity. In light of the fact that the fibril form of αS forms β-strands that assemble into in register parallel β-sheets this would suggest that the N-C terminal dimers would have to undergo conformational rearrangement to reach the final fibril form, thereby delaying or inhibiting the kinetics of fibril formation.

βS plays a role in the inhibition of αS aggregation but the mechanism by which this occurs and the stage in the aggregation pathway at which βS first interacts with αS has been unknown. We demonstrate that the monomer species of αS and βS interact directly with one another at specific sites suggesting that inhibition may begin at the very earliest stages of the fibril formation process. The molecular interactions and affinities obtained in the NMR PRE experiments described here support the view that early stages of aggregation versus inhibition may be due to a balance between conformational heterogeneity, specificity and affinity. Early stages of aggregation in αS may be promoted by sampling or searching conformational structures with weak transient affinities, including both non-specific head- to- head and weak specific head- to- tail dimers (Fig. 5A). In contrast early stages of inhibition may be favored by sampling only head- to- tail interactions with higher affinity and specificity within the αS/βS hetero-complex (Fig. 5B).

Head- to- tail interactions exist in both the homo- and hetero-dimer complexes, which strongly suggests that they may play a regulatory role on αS folding or misfolding. In light of the fact that the fibril state of αS assembles into in-register parallel cross-β-structure^46^,^47^, the NMR data suggests that head-to-tail αS/βS dimers would have to undergo conformational rearrangement to reach the final fibril form. This may thereby delay or inhibit the kinetics of fibril formation. We propose that the αS homo-complexes are more aggregation prone than αS/βS complexes for two reasons: first, αS can sample head- to- head interactions that are potentially aggregating promoting while αS/βS does not sample these; second, the non-propagating head- to- tail αS/αS complex has weaker, lower affinity interactions relative to the αS/βS complex suggesting that the conformational rearrangement required for fibril formation may be more facile for the αS/αS homo-complex.

Despite the fact that both the αS/αS homo-dimer and the αS/βS hetero-dimer sample head- to –tail interactions, there are notable differences in terms of the strength of the interactions and the range over which the interactions extend. For homodimers the interaction of the αS “hot spot” with the C-terminus of αS extends over ~15 residues, while the interaction of the αS “hot spot” with the C-terminus of βS extends over a broader range of residues from 105 to 134. In both cases the dimers are flexible and are able to probe a big surface area, possibly adopting multiple dimer conformations. In addition, the αS and βS C-termini are highly negatively charged and contain aromatic and hydrophobic residues suggesting that initial interactions are mediated by electrostatics, which are anchored and stabilized through the hydrophobic and aromatic interactions.

Using NMR PRE titration experiments we demonstrate that interactions between the C-terminus of βS and the N-terminus of αS are approximately 5 times stronger and more extensive than the interactions of the C-terminus of αS with its own N-terminus. This increased specificity and affinity may be attributed to two factors: a higher content of negative C-terminal charges thereby enhancing electrostatic interactions, and a higher proportion of proline residues thereby altering the conformational ensembles sampled by the C-terminus. NMR and ESI data indicate that the C-terminal of βS has a higher population of more extended species (Supplementary Fig. 1) and may therefore provide a more accessible surface area for interactions with the N-terminus “hot spot’ of αS. These differences suggest that small changes in the binding surface, even in these highly dynamic IDP complexes, can lead to substantial changes in interactivity that can modulate the pathway of protein aggregation versus inhibition.

In conclusion, our studies highlight that transient and weak interactions are important for protein recognition pathways of IDPs that lead to diseases such as amyloidosis where the proteins self-associate and propagate to highly ordered fibrils. Work by Radford *et al.*^48^ have shown that weak interactions are also important for folded proteins in directing aggregation versus inhibition. By performing NMR inter-chain PRE titration experiments we have identified and characterized the strength and affinity of transient interactions between αS and βS, both IDPs. As IDPs are highly flexible, binding affinities are likely to behave in a non-cooperative manner across the protein^49^. Our data support this view and highlight the variable nature of the binding affinities that result in aggregation promoting versus aggregation inhibiting configurations.

Knowledge about the distinct dissociation constants of different interaction regions provides a new framework for thinking about therapeutic intervention by providing direct information about which regions to target for small molecule intervention and about the conformational features that may be most effective at intervention. There have been some small molecule inhibitors that have targeted the N-terminus of αS but now based on our detailed molecular understanding of αS/βS interactions we can optimize the surface interactions to design novel inhibitors^10^,^11^,^12^,^50^,^51^,^52^. In addition, the powerful methods used here to identify the early stages of interaction of αS with βS can be extended to design inhibitors with biologics and can be applied even more widely to study other cross amyloid interactions, such as those between αS and amyloid- β-protein, or αS and tau, that have been shown to play a critical role in cross-seeding in neurodegenerative disease^48^,^53^,^54^,^55^,^56^.

## Methods

### Mutagenesis, expression and purification

Cysteine mutants of αS (A11C, T44C) and βS (A11C, T44C, A80C, A134C), were prepared by site-directed mutagenesis using AccuPrime pfx from Invitrogen. To obtain N-terminal acetylated forms of αS and βS proteins, co-expression with the NatB plasmid N-Acetyltransferase B was performed, as described previously^44^. Protein purification was performed according to previous protocols^57^. Similarly, MTSL spin label conjugation to cysteine mutants was performed using previously established protocols^35^.

### PRE experiments/controls

All NMR PRE experiments were performed in 10mM MES, pH 6, without addition of salt and with 10% D_2_O required for NMR experiments. NMR inter-chain PRE experiments were performed by mixing NMR blind ^14^N-MTSL labeled protein with NMR visible ^15^N unmodified protein^35^. Samples were prepared as follows: lyophilized samples of ^14^N-MTSL-cysteine mutants or ^15^N non-modified proteins (αS or βS) were separately dissolved. Samples were passed through a 100 kDa filter to remove higher order oligomers, and then concentrated using 3 kDa filters to be able to dilute the sample to a final concentration of 250 uM. Low sample concentration was chosen to minimize non-specific interactions. The total sample concentration was 500 uM, with 250 uM non-modified ^15^N protein and 250 μM ^14^N-MTSL labeled protein. All combinations of proteins were mixed in order to see all possible interactions. Diamagnetic samples were prepared by reducing samples with 10× excess of Ascorbic Acid and 5× buffer exchange using 3kDa cutoff filters from Millipore Inc. All the controls have similar patterns and are in the range of experimental error. Additionally, the pattern for the reduced diamagnetic control is consistent with the pattern for the mixture of ^14^N and ^15^N non-modified samples. The ^1^H-^15^N HSQC of the cysteine mutants with the reduced spin label did not disrupt the HSQC pattern of αS and βS.

All ^1^H-R_2_ measurements of paramagnetic and diamagnetic (reduced) samples were acquired on a 600 MHz Varian at 15 °C using previously published pulse sequence and protocols^33^,^35^. The inter-chain paramagnetic relaxation enhancement rate (PRE rate–Γ_2_) is the residue specific difference of the ^1^H-R_2_ values of the paramagnetic and diamagnetic samples. Increased ^1^H-R_2_ relaxation rates on the ^15^N visible chain indicates that the NMR blind ^14^N -MTSL labeled protein is in the proximity of specific residues in the NMR ^15^N labeled visible chain. Para- and diamagnetic ^1^H-R_2_ were analyzed and processed using nmrpipe^58^ and sparky^59^. For all experiments 10 relaxation delays were used: 12, 32, 104, 12, 124, 64, 48, 94, 64, 20 ms. Two data points (12 ms, 64 ms) were repeated in the experiment to obtain good statistics for the error analysis. Errors of Γ_2_ were calculated using error propagation, and errors were below 2 Hz. All of the interactions that were considered significant were at least 2 times higher than the mean value, and all of them were higher than the 3rd quartile and at least 8Hz.

### NMR PRE titration experiments

For NMR PRE titration experiments we used protocols described before^36^,^45^. Spin label sample concentrations were reduced to low volume >32 uL and were added to 350uL of 250uM ^15^N sample of either αS or βS; the changes in the sample volume were less that 10% of the overall sample volume. For αS titrations we used the following ratio of ^14^N- αS-44-MTSL labeled samples to ^15^N NMR visible samples: 0, 0.25, 0.5, 0.75, 1, 1.5, and in the case of βS we went up to ratio of 2. αS in this ratio exhibited shifts in the HSQC spectra, thus we removed this point from the analysis. We ran 6 data points ranging from 12–125 ms with the first point repeated twice for statistics. The PRE profiles during the titration did not show significant contributions from the non-interactive regions. The relaxation rates for the non-interactive NAC region for the titration ratio ^15^N/^14^N equal to 1.5 are well below 8 Hz.

### NMR PRE titration fitting and analysis

Titration curves of ^14^N-MTSL labeled protein with ^15^N protein were fit using equation (1):


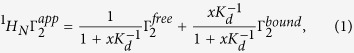


where *x* is the concentration of ^14^N-αS (T44C-MTSL) in solution, Γ_2_^free^–represents paramagnetic relaxation enhancement for free protein, and Γ_2_^bound^ represents the maximum observed saturation value. The fitting scheme was based on the papers by Bax^45^ and Clore^36^. We used a nonlinear regression model with three fitting parameters Γ_2,free_, Γ_2_^bound^ and *K*_*d*_. The χ-squared statistic that measures the difference between the observed and predicted increase in ^1^H_N_Γ_2_^app^ was optimized using the R statistics package minpack.lm, via the Levenberg-Marquardt algorithm^60^. The resulting fit was robust to small changes in Γ_2_^free^, K_D_, and Γ_2_^bound^ and provided us with residue specific K_D_ values between ^14^N-αS-44-MTSL and ^15^N-αS or ^15^N-βS. The *K*_*d*_ calculations were performed for residues whose 5^th^ titration point had PRE values higher that 15 Hz (Supplementary table 1&2). Results are summarized in the supplementary tables 1&2.

### Thioflavin T (ThT) aggregation assays

The experimental set up for measuring time dependent fibril formation with ThT has been previously described^57^. The conditions used for the ThT assay match the conditions of the NMR PRE experiments described earlier. 5–10 mg of lyophilized αS and βS was dissolved in 10 mM MES, pH 6, and centrifuged for 10 min. at 14000 rpm to remove big oligomers, and purified using size exclusion chromatography (Superdex 75 GL 10/300, from GE Healthcare Life Sciences). Protein was subsequently concentrated using 3kDa centrifugal units (Millipore Inc). The final concentration of αS and/or βS was 70 μM. Addition of βS to αS was performed in multiples of 70 μM. ThT fibrillation rates of the following samples were measured: (1) αS alone (2) βS alone; (3) ThT fluorescence of 1:1 the mixture αS + βS, and (4) ThT fluorescence of 1:5 the mixture of αS+5xβS. Each condition was repeated 3 times in 37 °C, with linear shaking in the presence of the Teflon beads. For each sample 3 curves are plotted on the figure to show the variability of the aggregation profiles.

## Additional Information

**How to cite this article**: Janowska, M. K. *et al.* Unveiling transient protein-protein interactions that modulate inhibition of alpha-synuclein aggregation by beta-synuclein, a pre-synaptic protein that co-localizes with alpha-synuclein. *Sci. Rep.* **5**, 15164; doi: 10.1038/srep15164 (2015).

## Supplementary Material

Supplementary Information

## References

[b1] Soto C. Unfolding the role of protein misfolding in neurodegenerative diseases. Nat Rev Neurosci 4, 49–60 (2003). 12511861 10.1038/nrn1007

[b2] Uversky V. N., Oldfield C. J. & Dunker A. K. Intrinsically disordered proteins in human diseases: Introducing the D(2) concept. Annu Rev Biophys 37, 215–246 (2008). 18573080 10.1146/annurev.biophys.37.032807.125924

[b3] de Lau L. M. & Breteler M. M. Epidemiology of Parkinson’s disease. Lancet Neurol 5, 525–535 (2006). 16713924 10.1016/S1474-4422(06)70471-9

[b4] Cooper A. A. *et al.* Alpha-synuclein blocks ER-Golgi traffic and Rab1 rescues neuron loss in Parkinson’s models. Science 313, 324–328 (2006). 16794039 10.1126/science.1129462 PMC1983366

[b5] Burre J., Sharma M. & Sudhof T. C. alpha-Synuclein assembles into higher-order multimers upon membrane binding to promote SNARE complex formation. Proc Natl Acad Sci USA 111, E4274–E4283 (2014). 25246573 10.1073/pnas.1416598111 PMC4210039

[b6] Murphy D. D., Rueter S. M., Trojanowski J. Q. & Lee V. M. Synucleins are developmentally expressed, and alpha-synuclein regulates the size of the presynaptic vesicular pool in primary hippocampal neurons. J Neurosci 20, 3214–3220 (2000). 10777786 10.1523/JNEUROSCI.20-09-03214.2000 PMC6773130

[b7] Wang L. *et al.* alpha-Synuclein Multimers Cluster Synaptic Vesicles and Attenuate Recycling. Curr Biol 24, 2319–2326 (2014). 25264250 10.1016/j.cub.2014.08.027 PMC4190006

[b8] Jiang Z., de Messieres M. & Lee J. C. Membrane remodeling by alpha-synuclein and effects on amyloid formation. J Am Chem Soc 135, 15970–15973 (2013). 24099487 10.1021/ja405993r PMC3859146

[b9] Lamberto G. R. *et al.* Toward the Discovery of Effective Polycyclic Inhibitors of alpha-Synuclein Amyloid Assembly. J Biol Chem 286, 32036–32044 (2011). 21795682 10.1074/jbc.M111.242958 PMC3173224

[b10] Meng X. Y., Munishkina L. A., Fink A. L. & Uversky V. N. Molecular Mechanisms Underlying the Flavonoid-Induced Inhibition of alpha-Synuclein Fibrillation. Biochemistry-Us 48, 8206–8224 (2009). 10.1021/bi900506b 19634918

[b11] Rao J. N., Dua V. & Ulmer T. S. Characterization of alpha-synuclein interactions with selected aggregation-inhibiting small molecules. Biochemistry-Us 47, 4651–4656 (2008). 10.1021/bi8002378 18366183

[b12] Lendel C. *et al.* On the Mechanism of Nonspecific Inhibitors of Protein Aggregation: Dissecting the Interactions of alpha-Synuclein with Congo Red and Lacmoid. Biochemistry-Us 48, 8322–8334 (2009). 10.1021/bi901285x 19645507

[b13] Horvath I. *et al.* Mechanisms of protein oligomerization: inhibitor of functional amyloids templates alpha-synuclein fibrillation. J Am Chem Soc 134, 3439–3444 (2012). 22260746 10.1021/ja209829m PMC3290101

[b14] Jones D. R., Moussaud S. & McLean P. Targeting heat shock proteins to modulate alpha-synuclein toxicity. Ther Adv Neurol Disord 7, 33–51 (2014). 24409201 10.1177/1756285613493469 PMC3886379

[b15] Breydo L., Wu J. W. & Uversky V. N. alpha-Synuclein misfolding and Parkinson’s disease. Bba-Mol Basis Dis 1822, 261–285 (2012). 10.1016/j.bbadis.2011.10.002 22024360

[b16] Uversky V. N. *et al.* Biophysical properties of the synucleins and their propensities to fibrillate: inhibition of alpha-synuclein assembly by beta- and gamma-synucleins. J Biol Chem 277, 11970–11978 (2002). 11812782 10.1074/jbc.M109541200

[b17] Park J. Y. & Lansbury P. T. Jr. Beta-synuclein inhibits formation of alpha-synuclein protofibrils: a possible therapeutic strategy against Parkinson’s disease. Biochemistry-Us 42, 3696–3700 (2003). 10.1021/bi020604a 12667059

[b18] Tsigelny I. F. *et al.* Dynamics of alpha-synuclein aggregation and inhibition of pore-like oligomer development by beta-synuclein. Febs J 274, 1862–1877 (2007). 17381514 10.1111/j.1742-4658.2007.05733.x

[b19] Hashimoto M. *et al.* Beta-synuclein regulates Akt activity in neuronal cells. A possible mechanism for neuroprotection in Parkinson’s disease. J Biol Chem 279, 23622–23629 (2004). 15026413 10.1074/jbc.M313784200

[b20] da Costa C. A., Masliah E. & Checler F. Beta-synuclein displays an antiapoptotic p53-dependent phenotype and protects neurons from 6-hydroxydopamine-induced caspase 3 activation: cross-talk with alpha-synuclein and implication for Parkinson’s disease. J Biol Chem 278, 37330–37335 (2003). 12867415 10.1074/jbc.M306083200

[b21] Lee D., Paik S. R. & Choi K. Y. Beta-synuclein exhibits chaperone activity more efficiently than alpha-synuclein. Febs Lett 576, 256–260 (2004). 15474047 10.1016/j.febslet.2004.08.075

[b22] Shaltiel-Karyo R. *et al.* Inhibiting alpha-synuclein oligomerization by stable cell-penetrating beta-synuclein fragments recovers phenotype of Parkinson’s disease model flies. Plos One 5, e13863 (2010). 21085664 10.1371/journal.pone.0013863 PMC2978097

[b23] Beyer K., Ispierto L., Latorre P., Tolosa E. & Ariza A. Alpha- and beta-synuclein expression in Parkinson disease with and without dementia. J Neurol Sci 310, 112–117 (2011). 21683963 10.1016/j.jns.2011.05.049

[b24] Rockenstein E. *et al.* Altered expression of the synuclein family mRNA in Lewy body and Alzheimer’s disease. Brain Res 914, 48–56 (2001). 11578596 10.1016/s0006-8993(01)02772-x

[b25] Hashimoto M., Rockenstein E., Mante M., Mallory M. & Masliah E. beta-Synuclein inhibits alpha-synuclein aggregation: a possible role as an anti-parkinsonian factor. Neuron 32, 213–223 (2001). 11683992 10.1016/s0896-6273(01)00462-7

[b26] Hashimoto M. *et al.* An antiaggregation gene therapy strategy for Lewy body disease utilizing beta-synuclein lentivirus in a transgenic model. Gene Ther 11, 1713–1723 (2004). 15483670 10.1038/sj.gt.3302349

[b27] Fan Y. *et al.* Beta-synuclein modulates alpha-synuclein neurotoxicity by reducing alpha-synuclein protein expression. Hum Mol Genet 15, 3002–3011 (2006). 16959793 10.1093/hmg/ddl242

[b28] Rivers R. C. *et al.* Molecular determinants of the aggregation behavior of alpha- and beta-synuclein. Protein Sci 17, 887–898 (2008). 18436957 10.1110/ps.073181508 PMC2327276

[b29] Zibaee S. *et al.* Sequence determinants for amyloid fibrillogenesis of human alpha-synuclein. J Mol Biol 374, 454–464 (2007). 17936783 10.1016/j.jmb.2007.09.039

[b30] Roodveldt C. *et al.* A Rationally Designed Six-Residue Swap Generates Comparability in the Aggregation Behavior of alpha-Synuclein and beta-Synuclein. Biochemistry-Us 51, 8771–8778 (2012). 10.1021/bi300558q 23003198

[b31] Taschenberger G. *et al.* Bs-synuclein aggregates and induces neurodegeneration in dopaminergic neurons. Ann Neurol 74, 109–118 (2013). 23536356 10.1002/ana.23905

[b32] Galvin J. E., Uryu K., Lee V. M. & Trojanowski J. Q. Axon pathology in Parkinson’s disease and Lewy body dementia hippocampus contains alpha-, beta-, and gamma-synuclein. Proc Natl Acad Sci USA 96, 13450–13455 (1999). 10557341 10.1073/pnas.96.23.13450 PMC23968

[b33] Clore G. M. & Iwahara J. Theory, practice, and applications of paramagnetic relaxation enhancement for the characterization of transient low-population states of biological macromolecules and their complexes. Chem Rev 109, 4108–4139 (2009). 19522502 10.1021/cr900033p PMC2825090

[b34] Tang C., Ghirlando R. & Clore G. M. Visualization of transient ultra-weak protein self-association in solution using paramagnetic relaxation enhancement. J Am Chem Soc 130, 4048–4056 (2008). 18314985 10.1021/ja710493m

[b35] Wu K. P. & Baum J. Detection of Transient Interchain Interactions in the Intrinsically Disordered Protein alpha-Synuclein by NMR Paramagnetic Relaxation Enhancement. J Am Chem Soc 132, 5546; PMCID: PMC3064441 (2010). 20359221 10.1021/ja9105495 PMC3064441

[b36] Fawzi N. L., Doucleff M., Suh J. Y. & Clore G. M. Mechanistic details of a protein-protein association pathway revealed by paramagnetic relaxation enhancement titration measurements. P Natl Acad Sci USA 107, 1379–1384 (2010). 10.1073/pnas.0909370107 PMC2824347 20080627

[b37] Clayton D. F. & George J. M. The synucleins: a family of proteins involved in synaptic function, plasticity, neurodegeneration and disease. Trends Neurosci 21, 249–254 (1998). 9641537 10.1016/s0166-2236(97)01213-7

[b38] Polevoda B. & Sherman F. N-terminal Acetyltransferases and Sequence Requirements for N-terminal Acetylation of Eukaryotic Proteins. J Mol Biol 325, 595–622 (2003). 12507466 10.1016/s0022-2836(02)01269-x

[b39] Sung Y. H. & Eliezer D. Residual structure, backbone dynamics, and interactions within the synuclein family. J Mol Biol 372, 689–707 (2007). 17681534 10.1016/j.jmb.2007.07.008 PMC2094134

[b40] Bertoncini C. W. *et al.* Structural characterization of the intrinsically unfolded protein beta-synuclein, a natural negative regulator of alpha-synuclein aggregation. J Mol Biol 372, 708–722 (2007). 17681539 10.1016/j.jmb.2007.07.009

[b41] Bourhim M., Kruzel M., Srikrishnan T. & Nicotera T. Linear quantitation of Abeta aggregation using Thioflavin T: reduction in fibril formation by colostrinin. J Neurosci Methods 160, 264–268 (2007). 17049613 10.1016/j.jneumeth.2006.09.013

[b42] Groenning M. *et al.* Binding mode of Thioflavin T in insulin amyloid fibrils. J Struct Biol 159, 483–497 (2007). 17681791 10.1016/j.jsb.2007.06.004

[b43] Peterson D. W., Zhou H. J., Dahlquist F. W. & Lew J. A soluble oligomer of tau associated with fiber formation analyzed by NMR. Biochemistry-Us 47, 7393–7404 (2008). 10.1021/bi702466a 18558718

[b44] Kang L. J. *et al.* N-terminal acetylation of alpha-synuclein induces increased transient helical propensity and decreased aggregation rates in the intrinsically disordered monomer. Protein Sci 21, 911–917; PMCID; PMC3403430 (2012). 22573613 10.1002/pro.2088 PMC3403430

[b45] Fitzkee N. C., Masse J. E., Shen Y., Davies D. R. & Bax A. Solution Conformation and Dynamics of the HIV-1 Integrase Core Domain. J Biol Chem 285, 18072–18084 (2010). 20363759 10.1074/jbc.M110.113407 PMC2878568

[b46] Vilar M. *et al.* The fold of alpha-synuclein fibrils. P Natl Acad Sci USA 105, 8637–8642 (2008). 10.1073/pnas.0712179105 PMC2438424 18550842

[b47] Comellas G. *et al.* Structured Regions of alpha-Synuclein Fibrils Include the Early-Onset Parkinson’s Disease Mutation Sites. J Mol Biol 411, 881–895 (2011). 21718702 10.1016/j.jmb.2011.06.026 PMC3157309

[b48] Karamanos T. K., Kalverda A. P., Thompson G. S. & Radford S. E. Visualization of Transient Protein-Protein Interactions that Promote or Inhibit Amyloid Assembly. Mol Cell 55, 214–226 (2014). 24981172 10.1016/j.molcel.2014.05.026 PMC4104025

[b49] Marsh J. A., Teichmann S. A. & Forman-Kay J. D. Probing the diverse landscape of protein flexibility and binding. Curr Opin Struc Biol 22, 643–650 (2012). 10.1016/j.sbi.2012.08.008 22999889

[b50] Lamberto G. R. *et al.* Structural and mechanistic basis behind the inhibitory interaction of PcTS on alpha-synuclein amyloid fibril formation. P Natl Acad Sci USA 106, 21057–21062 (2009). 10.1073/pnas.0902603106 PMC2795524 19948969

[b51] Mirecka E. A. *et al.* Sequestration of a beta-Hairpin for Control of alpha-Synuclein Aggregation. Angew Chem Int Edit 53, 4227–4230 (2014). 10.1002/anie.201309001 24623599

[b52] Fonseca-Ornelas L. *et al.* Small molecule-mediated stabilization of vesicle-associated helical alpha-synuclein inhibits pathogenic misfolding and aggregation. Nat Commun 5, 5857–5868 (2014). 25524885 10.1038/ncomms6857

[b53] Guo J. L. & Lee V. M. Y. Cell-to-cell transmission of pathogenic proteins in neurodegenerative diseases. Nat Med 20, 130–138 (2014). 24504409 10.1038/nm.3457 PMC4011661

[b54] Clinton L. K., Blurton-Jones M., Myczek K., Trojanowski J. Q. & LaFerla F. M. Synergistic Interactions between A beta, Tau, and alpha-Synuclein: Acceleration of Neuropathology and Cognitive Decline. J Neurosci 30, 7281–7289 (2010). 20505094 10.1523/JNEUROSCI.0490-10.2010 PMC3308018

[b55] Irwin D. J., Lee V. M. Y. & Trojanowski J. Q. Parkinson’s disease dementia: convergence of alpha-synuclein, tau and amyloid-beta pathologies. Nat Rev Neurosci 14, 626–636 (2013). 23900411 10.1038/nrn3549 PMC4017235

[b56] Kurosinski P., Guggisberg M. & Gotz J. Alzheimer’s and Parkinson’s disease-overlapping or synergistic pathologies? Trends Mol Med 8, 3–5 (2002). 11796255 10.1016/s1471-4914(01)02246-8

[b57] Kang L. J., Wu K. P., Vendruscolo M. & Baum J. The A53T Mutation is Key in Defining the Differences in the Aggregation Kinetics of Human and Mouse alpha-Synuclein. J Am Chem Soc 133, 13465–13470 (2011). 21721555 10.1021/ja203979j PMC3205953

[b58] Delaglio F. *et al.* Nmrpipe-a Multidimensional Spectral Processing System Based on Unix Pipes. J Biomol Nmr 6, 277–293 (1995). 8520220 10.1007/BF00197809

[b59] Goddard T. D. & Kneller D. G. SPARKY 3.

[b60] Elzhov T. M. & Katharine M. Spiess Andrej-Nikolai; Bolker, B. minpack.lm: R interface to the Levenberg-Marquardt nonlinear least-squares algorithm found in MINPACK, plus support for bounds <https://cran.r-project.org/web/packages/minpack.lm/minpack.lm.pdf>, (2013) (Date of access:09/04/2015).

