# Supplementary material for: Unveiling transient protein-protein interactions that modulate inhibition of alpha-synuclein aggregation by beta-synuclein, a pre-synaptic protein that co-localizes with alpha-synuclein
Source: Sci Rep. 2015 Oct 19;5:15164. doi: 10.1038/srep15164 (PMC4609965; doi:10.1038/srep15164)
Supplement: Supplementary Information [file srep15164-s1.pdf]

Supplementary information

Unveiling transient protein-protein interactions that modulate inhibition of alpha-synuclein aggregation by beta-synuclein, a pre-synaptic protein that co-localizes with alpha-synuclein.

Maria K. Janowska, Kuen-Phon Wu and Jean Baum

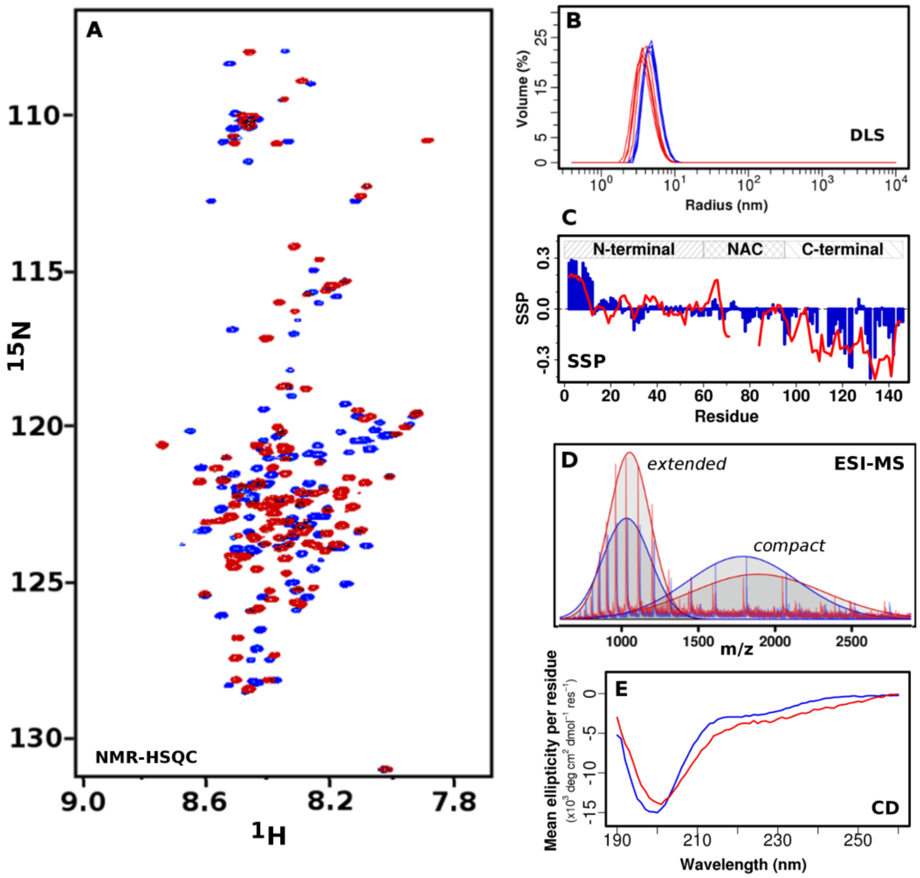

Supplementary Fig.1. Comparison of monomer conformational features of acetylated αS and acetylated βS**.** (A) ^{1}H-^{15}N-HSQC spectra of αS and βS at 15°C and pH 6. (B) DLS spectra showing that αS and βS both have hydrodynamic radii in the range of 5-6 nm. No oligomers were detetected and proteins are monomeric (>99%). (C) SSP indicates that N-terminal acetylation induces transient N-terminal helix formation in βS that is similar to acetylated αS; the transient helix in βS spans residues 1 to 12. (D) ESI-

MS shows that both αS and βS are 100% acetylated and that both proteins can exist in compact and extended conformations (indicated by fitting two Gaussians). αS has a 59% compact/ 41% extended ratio while βS has a 46% compact/ 54% extended ratio. (E) Circular Dichroism spectra indicate that both αS and βS have very similar profiles consistent with unfolded nature of the protein.

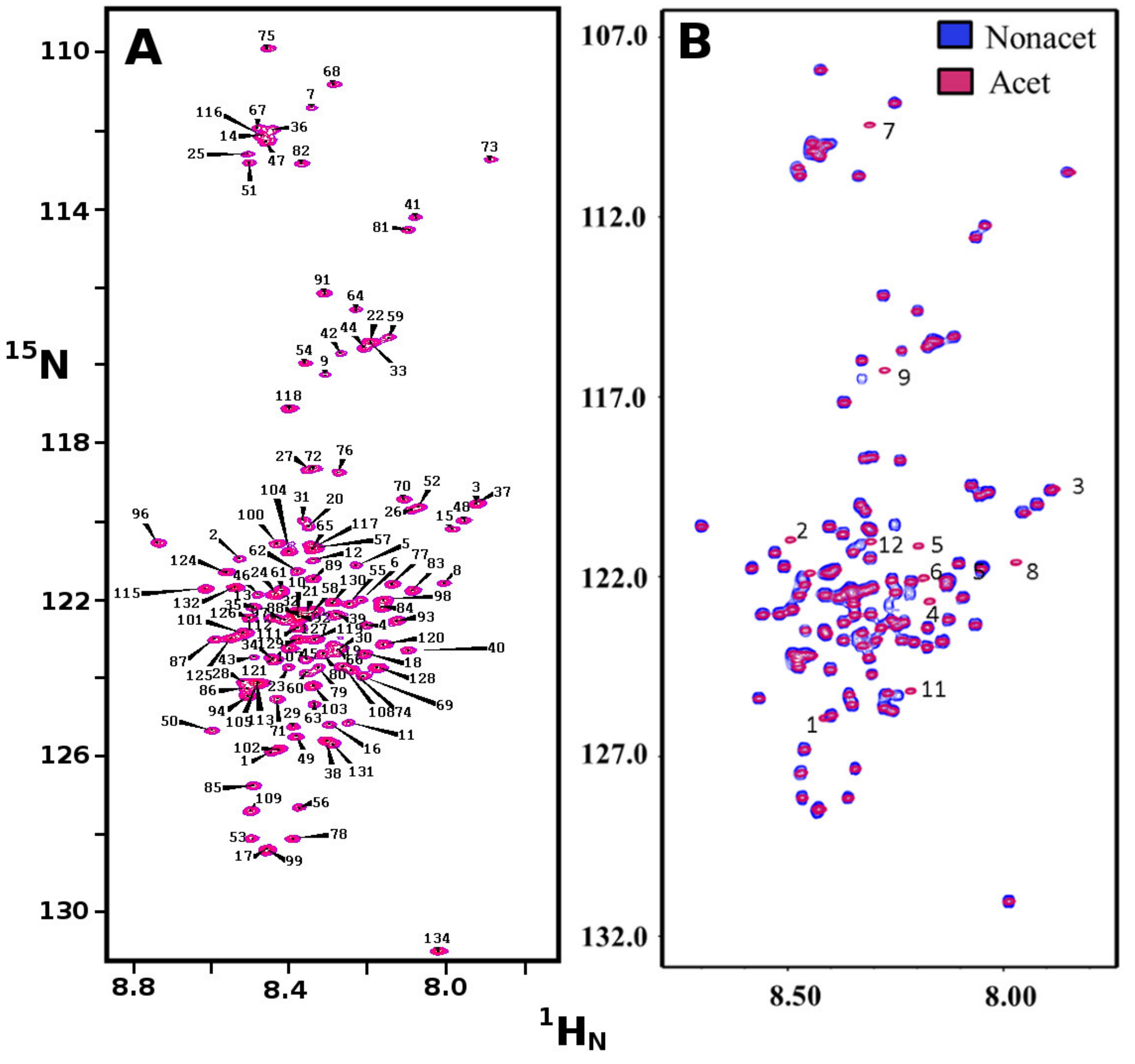

Supplementary Figure 2. (A) ^{1}H-^{15}N HSQC spectrum of acetylated βS. Sequence-specific resonance assignments are indicated with labels. (B) ^{1}H-^{15}N HSQC overlay of non-acetylated (blue) and acetylated (pink) βS.Changes in positions of the peaks due to acetylation arise for the first 12 residues, and are labeled on the spectrum with the corresponding residue number.

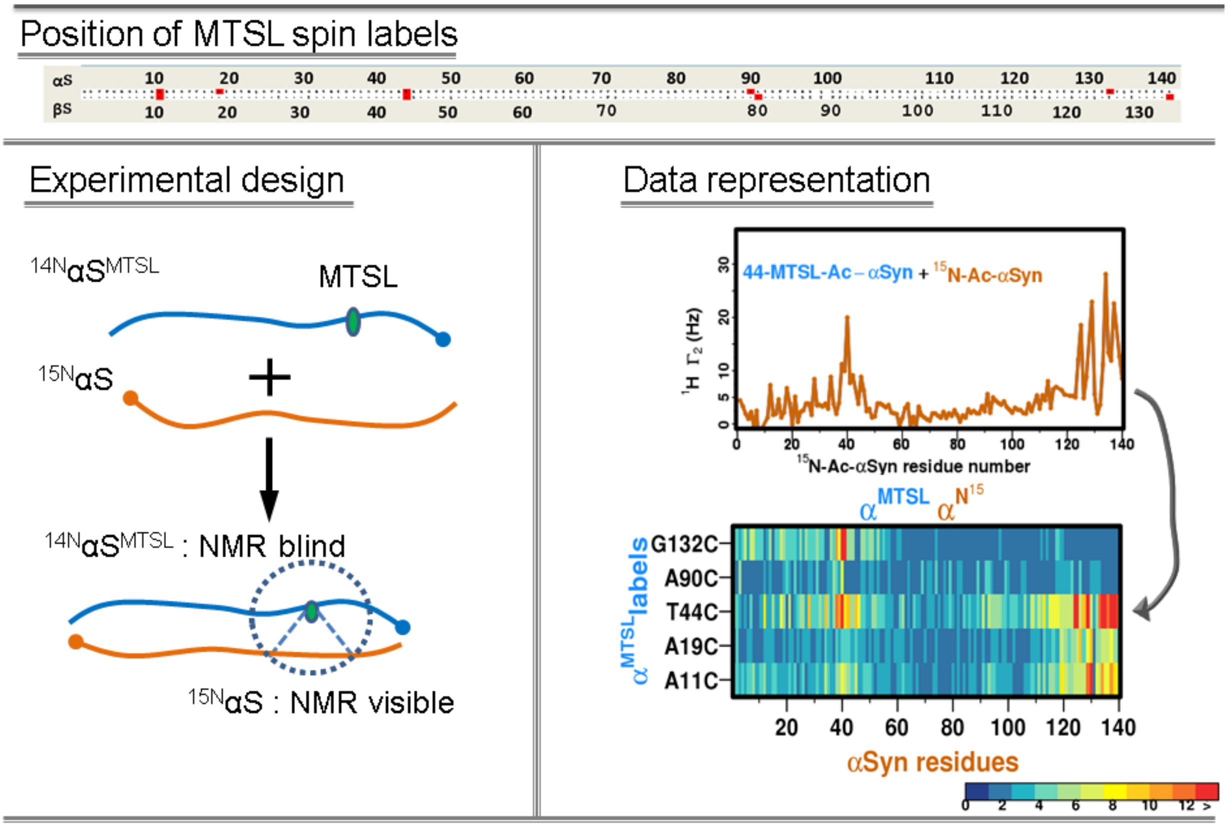

Supplementary Fig. 3. Schematic of experimental design for NMR PRE experiments. (A) 4 spin labels were introduced on each of the chains of the proteins, indicated by red box in the sequence of the protein. A->C and T->C mutants were selected in order to minimize effects of mutations. (B) Two different species are mixed in a 1:1 ratio: the NMR visible (^{15}N protein) and the NMR invisible MTSL spin labeled ^{14}N -protein. If the spin label is in the proximity to the ^{15}N labeled chain, the relaxation rates are increased (C) Example of the data representation. The top plot shows the relaxation enhancement rates (PRE rate - Γ) detected on the NMR visible chain (^{15}N). The lower plot shows the interactions in a pseudo contact map representation with the four spin labeled positions mapped against the 15N residues in the protein. The contact map is represented as a heat map with the color index shown in the inset.

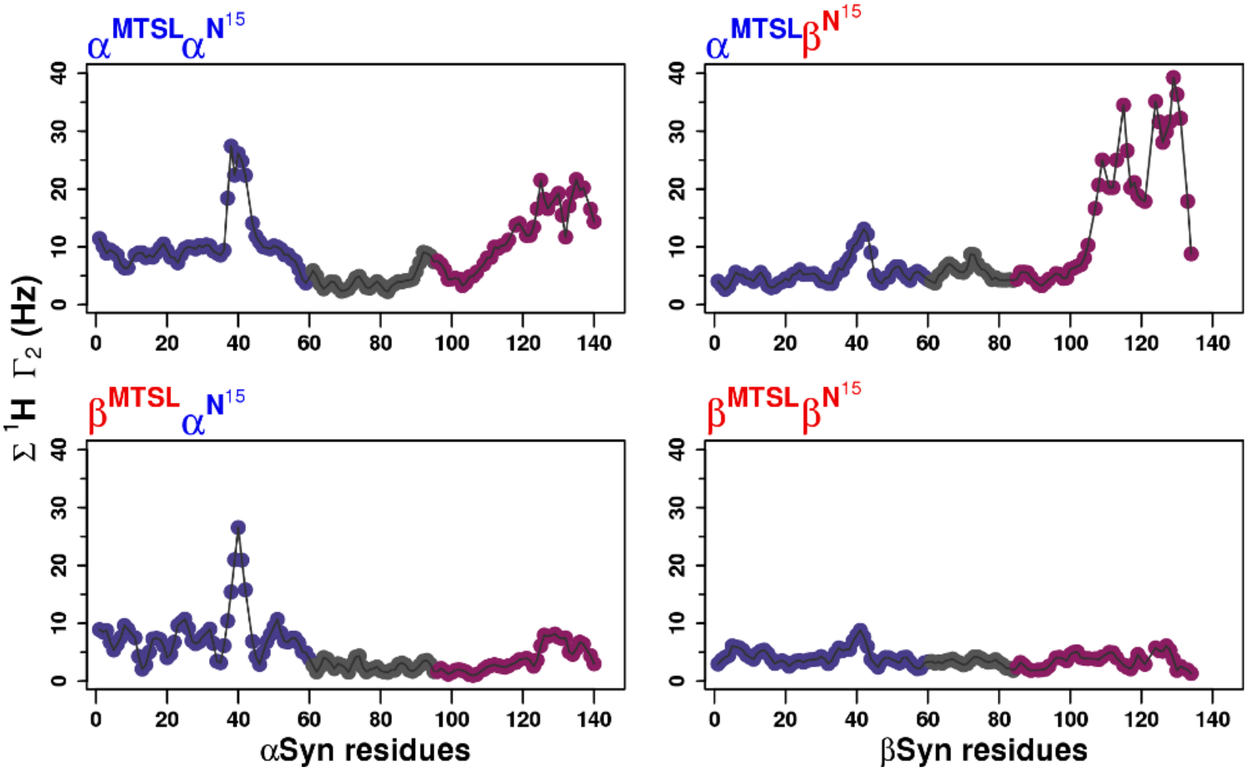

Supplementary Fig.4. Interactive regions of dimeric complexes. Summation of the PRE rates as a function of residue indicate that (A) in αS homo-dimeric complexes interactions occur at the N and C-termini. (B) In βS/αS hetero-dimer complexes, interactions with αS are mediated primarily through the C-terminus. (C) In the αS/βS hetero-dimer interactions are mediated by the αS N-terminus. (D) in βS homo-dimer there are essentially no interactions . In all plots N-terminus is plotted violet, NAC is grey and C-terminus is pink.

Supplementary Table 1. K_D values for αS/αS homodimer as fitted χ-squared statistic using equation (1).

**α/α homo-dimer**			
**residue number**	**K_D [μM]**	**Γ_2^{bound} [Hz]**	**Γ_2^{free} [Hz]**
119	151.5 ± 22.6	24.8±1.4	0.1±0.4

124	269.7± 115.3	26.6± 5.5	-0.1±0.9
125	397.8±143.7	56± 11.5	-0.6±1.1
126	87.1 ± 59.4	33.9± 6.9	-0.4±3.5
129	157.4±53.2	41.2± 5.4	-0.2±1.7
130	719.7±216.1	128.1±26.1	0.4±1.1
135	241.2± 45.4	29.2±2.5	0.1±0.5
137	1219.8+-/858.6	133.9±72.7	0.9±1.2
139	934.3±532.1	75.5±31.2	0.6±0.8

Supplementary Table 2. K_D values for αS/βS hetero-dimer as fitted using χ-squared statistic.

**α/β hetero-dimer**			
**residue number**	**K_D [μM]**	**Γ_2^{bound} [Hz]**	**Γ_2^{free} [Hz]**
111	36.8±34.2	22.5±3.5	-0.2±3.6
112	107.8±39.4	22.6±2.4	-0.3±1.4
115	348.8±111.1	60.7±9.1	0±1.7
116	84.8±29.4	19.2±1.7	-0.2±1.2
118	141.3±34	23.4±1.8	-0.3±0.9
119	140.5±88.4	35.3±7.3	-0.9±3.5
120	338.3±166.7	55.3±12.7	0.1±2.4
121	187±56	40.1±4.4	-0.5±1.6
124	54.7±31.5	29.3±3.6	-0.2±3.1
125	98.3±31.9	28±2.5	-0.3±1.6

126	105.3±21.2	29.9±1.7	-0.2±1
127	36.8±34.2	22.5±3.5	-0.2±3.6
128	90.7±56.2	38.2±6.3	0±4.2
129	133.5±63.6	40.7±6.2	-0.7±3.1
131	80.7±21.8	32.3±2.2	0.1±1.6
133	142.6±20.1	31.2±1.4	-0.2±0.7

Supplementary Methods

1. NMR experiments. Assignments on βS and αS were performed using the protocol described elsewhere.[1] Backbone atom assignments were established in acetylated βS for all residues except for the prolines. Acetylation of the protein facilitated assignment of the first few residues, which are highly flexible in the non-acetylated form of the protein. Experiments were performed on 350uM ^{15}N and ^{13}C labeled sample with 10%D_2O in 10mM MES buffer pH 6 with 100mM NaCl. Secondary structure propensities were extracted using the SSP program by Julie Forman-Kay.[2] If the values are positive then this region has helical propensities, if the values are negative then the beta-sheet propensities are more pronounced.

2.Electrospray ionization mass spectroscopy (ESI-MS). ESI-MS experiments were performed in order to determine the population distributions of the monomeric ensembles of αS and βS using methods described previously.[3] Samples were prepared in 10mM Ammonium Acetate, pH 6 in final concentration 50uM, by using 100 kDa and 3kDa filters.

3.Dynamic light scattering (DLS). DLS measurements were carried out using a Zetasizer Nano ZS (Malvern Instruments, UK). Data was collected using a 3 mW He-Ne laser light at a 633 nm wavelength back scattered light at an angle of 173°. Autocorrelation functions were determined from 6 correlation

functions, with an acquisition time of 10 s for one correlation function. The sample concentration was 200uM.

4.Negative straining TEM. Fibrils were visualized using a JEM-100CXII manufactured by JEOL. Negative staining TEM was performed using a single droplet procedure[4] at ambient temperature. Micrographs were recorded at a magnification of 100,000. All of the chemicals are purchased from Sigma.

1 Kang, L. J., Wu, K. P., Vendruscolo, M. & Baum, J. The A53T Mutation is Key in Defining the Differences in the Aggregation Kinetics of Human and Mouse alpha-Synuclein. *J Am Chem Soc* **133**, 13465-13470, doi:Doi 10.1021/Ja203979j (2011).

2 Marsh, J. A., Singh, V. K., Jia, Z. C. & Forman-Kay, J. D. Sensitivity of secondary structure propensities to sequence differences between alpha- and gamma-synuclein: Implications for fibrillation. *Protein Sci* **15**, 2795-2804, doi:Doi 10.1110/Ps.062465306 (2006).

3 Moriarty, G. M., Minetti, C. A., Remeta, D. P. & Baum, J. A revised picture of the Cu(II)-alpha-synuclein complex: the role of N-terminal acetylation. *Biochemistry-Us* **53**, 2815-2817, doi:10.1021/bi5003025 (2014).

4 Horne, R. W. & Cockayne, D. J. H. Negative Staining. *Micron Microsc Acta* **22**, 319-319, doi:Doi 10.1016/0739-6260(91)90050-A (1991).
